# Magnetically Driven Micromachines Created by Two-Photon Microfabrication and Selective Electroless Magnetite Plating for Lab-on-a-Chip Applications

**DOI:** 10.3390/mi8020035

**Published:** 2017-01-24

**Authors:** Tommaso Zandrini, Shuhei Taniguchi, Shoji Maruo

**Affiliations:** 1 Dipartimento di Fisica, Politecnico di Milano, Piazza Leonardo da Vinci 32, 20133 Milano, Italy; tommaso.zandrini@polimi.it or tommaso.zandrini@gmail.com; 2 Department of Mechanical Engineering, Yokohama National University, 79-5 Tokiwadai, Hodogaya-ku, Yokohama 240-8501, Japan; taniguchi-shuuhei-zj@ynu.jp

**Keywords:** two-photon microfabrication, two-photon polymerization (2PP), femtosecond lasers, electroless plating, magnetite plating, magnetic, micromachine, microrotors

## Abstract

We propose a novel method to fabricate three-dimensional magnetic microparts, which can be integrated in functional microfluidic networks and lab-on-a-chip devices, by the combination of two-photon microfabrication and selective electroless plating. In our experiments, magnetic microparts could be successfully fabricated by optimizing various experimental conditions of electroless plating. In addition, energy dispersive X-ray spectrometry (EDS) clarified that iron oxide nanoparticles were deposited onto the polymeric microstructure site-selectively. We also fabricated magnetic microrotors which could smoothly rotate using common laboratory equipment. Since such magnetic microparts can be remotely driven with an external magnetic field, our fabrication process can be applied to functional lab-on-a-chip devices for analytical and biological applications.

## 1. Introduction

There is a growing need for three-dimensional (3D) printing techniques, such as fused deposition modeling (FDM) [1,2], inkjet modeling [3,4], and stereolithography [5,6], for the fabrication of arbitrary, complicated 3D structures. Above all, one stereolithography technique, two-photon microfabrication, allows higher resolution than any other 3D printing technique, resulting in it being particularly suitable for the integration of micro-components inside lab-on-a-chip devices. Specifically, two-photon microfabrication enables the fabrication of 3D microstructures with down-to-100-nm resolution via direct laser writing using a femtosecond pulsed laser beam. Thanks to this ultrahigh resolution, it has been applied in a wide range of fields, including the production of photonic crystals [7], lab-on-a-chip devices [8], bioscaffolds [9,10], micromachines [11,12], and so forth.

In general, the microstructures produced by two-photon microfabrication are made from photosensitive materials such as photopolymers [13], photoresists [14], biopolymers [15], and hybrid materials containing surface-modified nanoparticles [16]. In the past few years, as a method to activate or functionalize polymeric microstructures after their fabrication, electroless plating has attracted much attention, for its ability to metallize nonconductive materials, typically polymers [17,18,19,20]. We also have focused on this method, and fabricated metallized 3D movable microparts using electroless copper plating [18]. The created microparts were successfully driven by scanning a low-power laser beam [12].

On the other hand, magnetic driving is gaining interest for its simplicity and versatility: the fabrication of movable magnetic microparts has been reported by Tian et al. using a ferropolymer [21], and by Wang et al. employing electroless plating of a Ni–P alloy [22]. The latter produced a microturbine, which, according to the authors, should have better mechanical performances with respect to the ferropolymer, but is free to move without constraints.

Here, as a different plating technique, we propose a highly selective method to fabricate magnetic microparts with the combination of two-photon microfabrication and electroless magnetite plating. Polymeric microparts were successfully coated with smooth and even plating magnetite film by modifying various experimental conditions such as the bath temperature, pH, and metal ion concentration. In addition, energy dispersive X-ray spectrometry (EDS) and the driving experiment of microrotors clarified the magnetic properties of these microparts and highlighted their importance.

## 2. Materials and Methods

To prepare an acrylic resin suitable for electroless plating, we mixed two acrylic monomers and a photoinitiator, respectively tris (2-hydroxyethyl) isocyanurate triacrylate (SR-368, Sartomer Japan Inc., Yokohama, Japan), trimethylolpropane tri-acrylate (SR-399, Sartomer Japan Inc.), and ethyl-2,4,6-trimethylbenzoylphenyl-phosphinate (Lucirin TPO-L, BASF Japan Ltd., Tokyo, Japan) [17]. The resin consists of equal parts SR-368 and SR-399 with 3 wt % Lucirin TPO-L (BASF Japan Ltd.). A methacrylic resin that cannot be metallized by electroless plating was also prepared by mixing ethoxylated (2) bisphenol A dimethacrylate (SR-3489, Sartomer Japan Inc.) with 3 wt % Lucirin TPO-L (BASF).

In our fabrication system, a mode-locked Ti:Sapphire laser (Mira 900-F, Coherent, Santa Clara, CA, USA, wavelength 752 nm, repetition rate 76 MHz, pulse width 200 fs) is used to induce two-photon-absorbed photopolymerization. The laser is equipped with a galvano-scanner system (Cambridge Technology M2 scanners, Novanta Japan Corp., Tokyo, Japan) to deflect the beam direction in two dimensions, and it is then focused using an oil-immersion objective lens with a numerical aperture of 1.4. The objective lens is scanned along the optical axis with a piezoelectric actuator (P-725.2CD, PI Japan Co. Ltd., Tokyo, Japan), so that 3D microstructures are made on a glass substrate that had been modified with (3-methacryloxy-propyl) tri-methoxysilane to promote the adhesion of the polymer. The typical power and scanning velocity of the laser beam were 100 mW and 100 m/s. After fabrication, the microstructures were washed in a solvent to remove un-solidified liquid resin.

**Figure 1:**
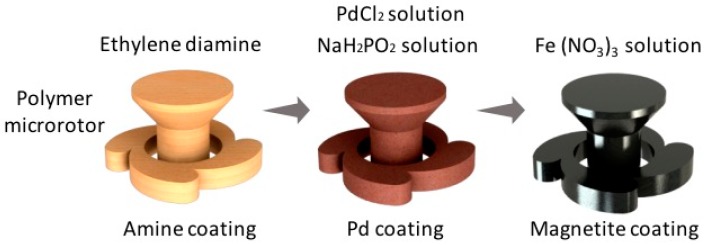
Electroless magnetite plating of a polymeric microrotor produced by two-photon microfabrication. Magnetite deposition on a polymeric microstructure can be realized through amine coating, Pd coating, and magnetite coating.

To coat the polymeric structures, we used an electroless plating method combined with previously reported site-selective Pd catalyst absorption process [18] and Fe_3_O_4_ deposition process [23], as shown in Figure 1. After the fabrication using the acrylic resin, the microstructures are immersed in a 20% (by vol.) solution of ethylene diamine (EDA, Wako Pure Chemical Industries, Ltd., Tokyo, Japan) in ethanol for 30 min, followed by three 1 min rinses in deionized water. The amine-coated samples are then submerged in an aqueous solution composed of 0.1 g/L PdCl_2_ and 0.1 mL/L of concentrated HCl for 15 min. The samples are rinsed in water twice for 1 min each before being placed in an 85 °C solution of 0.1 M NaH_2_PO_2_ for 10 min. Following that, the samples are washed in water three times for 1 min each. The final step is to dip the samples into the 70 °C iron oxide solution under stirring for 10 min. This solution consists of 0.01 M Fe(NO_3_)_3_ 9H_2_O and 0.03 M dimethylamine-borane (DMAB, Sigma-Aldrich Co. LLC., St. Louis, MI, USA) which act as metal salt and reducing agent, respectively. After deposition of magnetic materials, the samples are rinsed in water, and then dried in air.

Electroless plating reaction generally depends on some parameters such as bath temperature, pH, and metal ion concentration. In addition, these parameters have effects on other parameters reciprocally; we then experimentally examined optimal conditions to get higher quality plating film. Table 1 shows experimental conditions of electroless plating for 10 min.

In our experiments, we observed that, when the bath temperature is relatively high, the induced rapid nucleation made the surface of the magnetic micro-structure irregularly rough and uneven. On the other hand, a homogeneous surface can be obtained with a lower bath temperature, as we have shown in [24]. As a result of our optimization process, magnetic microstructures with a smooth and even plating film could be sophisticatedly fabricated with high reproducibility by adjusting different parameters, particularly bath temperature, as indicated in the third column of Table 1. The surface roughness (Ra) of a magnetite deposited microstructure was measured 0.1 µm by using a 3D Laser Scanning Confocal Microscope (VK-X250, KEYENCE Corp., Osaka, Japan). The thickness of the magnetite coating depends on the bath temperature. Typical thickness of the magnetite coating layer was measured at 1.3 µm with a bath temperature of 70 °C by comparing the size of microstructures before and after magnetite electroless plating with an optical microscope.

## 3. Results and Discussion

### 3.1. Elemental Analysis of Magnetic Microstructures Using Energy Dispersive X-ray Spectrometry (EDS)

**Figure 2:**
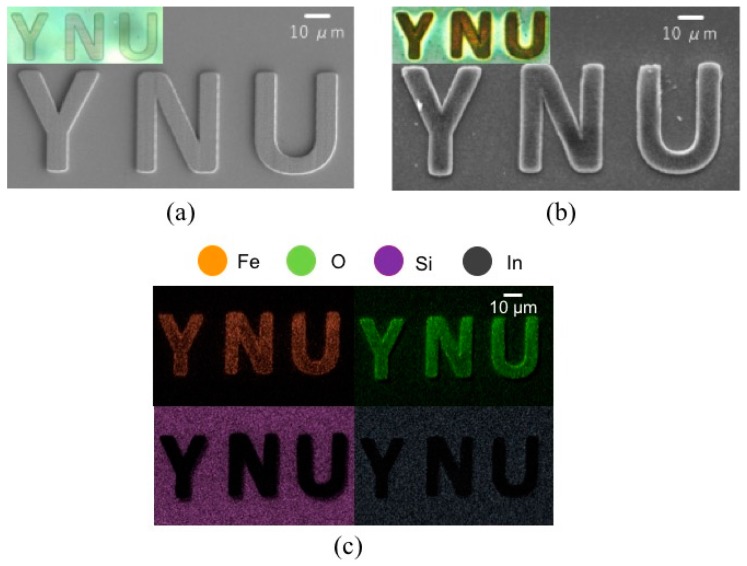
Experimental results of magnetite coating of polymeric microstructures made from the acrylic resin. (**a**,**b**) Scanning electron microscopy (SEM) image and optical image of the microstructures made from acrylic resin before and after electroless plating; (**c**) Element mappings of the magnetic microstructures (orange: iron, green: oxygen, violet: silicon, gray: indium).

**Figure 3:**
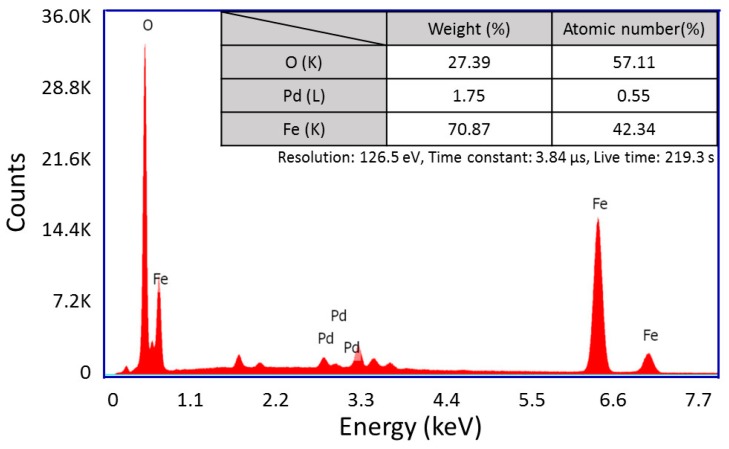
Energy dispersive X-ray spectrometry (EDS) spectra of the nanoparticles we prepared. Inserted table shows percent by weight and number of atoms for each element.

After the electroless plating of polymeric microstructures made from acrylic resin, the color variation from transparent to dark brown visually showed the magnetite deposition of these, as visible in Figure 2a,b. Then, to specifically confirm the presence of iron oxide in the plating film, we performed an elemental analysis on the magnetic microstructure. It was observed by a scanning electron microscopy (SEM, JSM-7001, JEOL Ltd., Tokyo, Japan), and some element mappings were obtained by an X-ray microanalysis (TEAM^TM^ EDS Analysis system, EDAX Business Unit AMETEK Co., Ltd., Tokyo, Japan). As a result, it was clear that iron oxide nanoparticles were deposited onto the microstructure made from acrylic resin site-selectively, as shown in Figure 2c. Since we used an indium tin oxide (ITO)-coated substrate (Hiraoka Glass Industry. Co., Ltd., Osaka, Japan, 0.4 mm × 30 mm × 40 mm) as a glass substrate, silicon and indium particles were detected outside the magnetic microstructure. As shown in Figure 3, oxygen had a strong peak because it was included not only in iron oxide particles but also in the ITO substrate. In addition, little impurities were detected in the EDS spectrum.

### 3.2. Selective Magnetite Deposition on Polymer Microstructures

**Figure 4:**
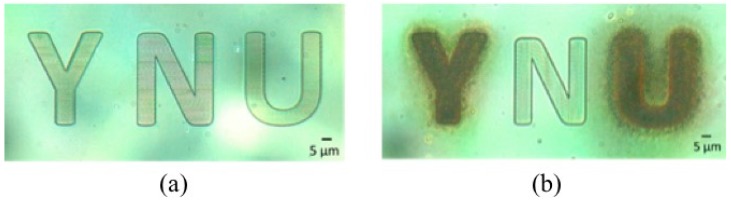
Optical images of selective magnetite coating of polymeric microstructures. (**a**,**b**) Optical images of the microstructures made from acrylic and methacrylic resin before and after electroless plating. The polymeric microstructure “N” made from methacrylic resin was not coated with magnetic materials.

To demonstrate the selective deposition of magnetic materials, we fabricated microstructures made from both acrylic and methacrylic resins. In Figure 4, the polymeric microstructure “N” was made from methacrylic resin, while “Y” and “U” were made from acrylic resin. After electroless plating, only the “N” was not coated with magnetic materials as shown in Figure 4b. This demonstrates that the methacrylic resin did not undergo amine coating, resulting, in our case, in selective magnetite deposition of the sole acrylic parts. In this experiment, the process time of electroless plating was longer than that of the optimal condition in order to show the selective magnetite coating clearly. For this reason, even though debris of magnetic materials was deposited around the microstructures made from the acrylic resin, the microstructure made from methacrylic resin was not coated with magnetic materials. This demonstrates the effectiveness of the selective magnetite coating using two kinds of resins.

**Figure 5:**
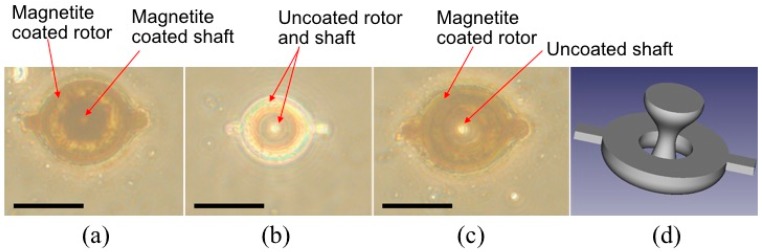
Experimental demonstration of selective magnetite coating of microrotors. (**a**–**c**) Optical images of fully coated acrylic rotor and shaft, uncoated methacrylic rotor and shaft, and selectively coated acrylic rotor with uncoated methacrylic shaft. Scale bar is 25 µm; (**d**) 3D CAD model of the microrotor.

In addition, we fabricated polymeric microrotors by employing both resins. As shown in Figure 5c, we succeeded in making a magnetite-coated acrylic microrotor with an uncoated methacrylic shaft. The internal brown circular part in Figure 5a is the optical image of the magnetite-coated shaft. On the other hand, the center white circular part in Figure 5c is the optical image of the uncoated shaft through which the transmitted light can pass. The uncoated shaft with the methacrylic resin was useful to reduce the friction and magnetic attraction between the rotor and shaft.

### 3.3. Remote Driving of Magnetite-Coated Microrotors

**Figure 6:**
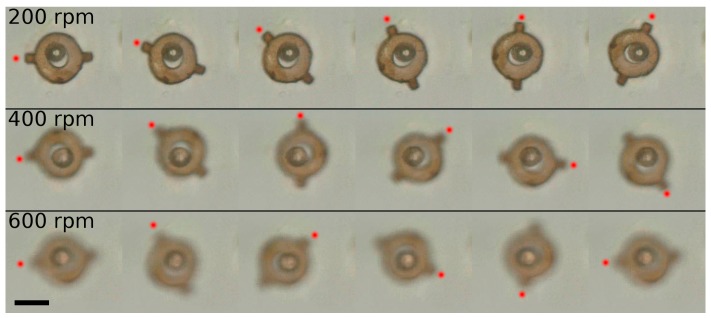
Subsequent frames recorded at 50 fps with stirrer rotation speed set to 200 rpm (first row), 400 rpm (second row), and 600 rpm (third row). Rotation is clockwise, red dots added manually as a visual hint. Scale bar is 20 µm. A short video of rotation at different speeds is available in Video S1.

The driving experiment was performed on a common laboratory magnetic stirrer placed on an optical microscope stage, with rotors immersed in a solution of water with 0.1% of Triton X-100 in the volume. In this setup, permanent magnets attached to a motor inside the stirrer gave a magnetic driving force to rotate the magnetite-coated microrotors. Smooth rotation was observed at rotational speeds from 140 to 600 rpm. Recording at higher rotational speeds has not been performed due to the limited frame rate of the camera and to mechanical vibrations introduced by the stirrer on the microscope stage, rather than due to a physical limit of our microrotors. In Figure 6, subsequent frames of rotation at 200, 400 and 600 rpm are shown, recorded at 50 fps. A short video of the rotation at different speeds is visible in Video S1.

Rotating components can be used for many applications in a microfluidic circuit, for example as micromixers or micropumps. The setup employed in this experiment is commonly available in every chemistry or biology laboratory, making this driving technique extremely simple and affordable for users without any experience in laser fabrications and microfluidics. Compared to the optical driving of microrotors and microelements in general, there is no need for a complex and expensive laser setup, eliminating the need for beam delivery and alignment, as well as user training. Moreover, multiple rotors can be moved simultaneously with the same setup.

## 4. Conclusions

We proposed a novel fabrication process for 3D magnetic microparts using two-photon microfabrication and selective electroless magnetite plating. In our method, electroless plating of the polymeric microstructures produced by two-photon microfabrication can provide sophisticated 3D magnetic microparts. In addition, we also experimentally investigated the optimal electroless plating conditions for coating the microstructures with a uniform layer of magnetite particles. With the same conditions, magnetic microparts were successfully fabricated. The plating film obtained by our process was analyzed by EDS, so that iron oxide nanoparticles were deposited onto the polymeric microstructure site-selectively. Driving experiments using a simple magnetic stirrer were performed, succeeding in the smooth rotation of magnetized microrotors around an uncoated shaft at a high rotation speed, demonstrating our capability to fabricate movable but constrained parts magnetized by electroless plating. In the near future, this fabrication process is expected to be applied to functional lab-on-a-chip devices for analytical and biological applications.

## Figures and Tables

**Table 1:** Experimental conditions of electroless plating.

Coating Parameter	Experimental Range	Optimal Range
Bath temperature (°C)	20–80	60–70
pH	3.0–5.0	3.5–4.5
Fe^3+^ (mol/L)	0.001–10.0	0.01–1.0

## Supplementary Materials

The following is available online at www.mdpi.com/2072-666X/8/2/35/s1, Video S1: Rotation of a magnetic microrotor at different speeds.
